# Treatment perspective after failed open reduction of congenital hip dislocation. A systematic review

**DOI:** 10.3389/fped.2023.1146332

**Published:** 2023-08-09

**Authors:** Sophie Merckaert, Pierre-Yves Zambelli

**Affiliations:** Unit of Pediatric Orthopedics, Department of Women-Mother – Child’s Care, Centre Hospitalier Universitaire Vaudois (CHUV), Lausanne, Switzerland

**Keywords:** avascular necrosis of the femoral head, congenital hip dislocation, DDH, failed open reduction, revision

## Abstract

**Background:**

Failure of open reduction of developmental hip dislocation is a serious complication and revision surgery appear to be technically demanding with high complication rates. Little attention has been given in literature to patients in whom open reduction of developmental hip dislocation has failed. We present a systematic review about current perspectives and timing when to perform surgical revision after failed open reduction of developmental hip dislocation in children.

**Methods:**

Following the recommendations of the “Preferred Reporting Items for Systematic Reviews and Meta-Analyses” (PRISMA) statements we performed a comprehensive search of the PubMed and Google Scholar bibliographic database in order to select all studies published between 1980 and 2022. Studies were screened for the reasons for failure of open reduction, timing when revision surgery was performed, and for the surgical techniques used for revision.

**Results:**

A total of 10 articles including 252 patients and 268 hips has been recorded. The most common causes of re-dislocation after open reduction are inadequate exposure and failure to release the obstructing soft tissues inside and around the hip. In 90% of the cases the anterolateral approach was performed for revision surgery. Avascular necrosis occurred in 5%–67% of cases and was the most encountered complication.

**Conclusion:**

Redislocation of developmental hip dislocation after an open reduction has poor long-term outcomes mainly due to a high rate of avascular necrosis of the femoral head. It is mandatory to obtain a stable reduction at the second surgery combining soft tissue release, capsulorrhaphy, pelvic and femoral osteotomies.

## Introduction

Developmental dysplasia of the hip (DDH) is defined as an insufficient acetabular coverage of the femoral head and can range from mild dysplasia to total hip dislocation (1).

The incidence of DDH per 1,000 live births ranges from 0.06 to 76 with significant variability between and within racial groups and depending on geographic location (2).

It is thought that approximately one in 1,000 children is born with a dislocated hip (3).

The treatment aims to achieve a concentric reposition, retention, and maturation of the hip (4, 5). Predictors of treatment success are timing at treatment initiation and severity of the dislocation. Indeed, early diagnosis and treatment of congenital hip dislocation are essential for a physiologic maturation of the hip joint and to prevent disability later in life (6, 7).

In patients with an early diagnosis within the first 6 months of life, treatment is essentially functional and involves the use of dynamic harness for dysplastic hips and orthoses for in unstable hips until maturation (8–10).

For dislocated hips the current gold standard is closed reduction under fluoroscopic guidance and subsequent spica cast immobilization under general anesthesia (11, 12).

Nevertheless, even in case of early treatment, the successful rate of closed reduction for developmental dislocation of the hip is highly variable and ranges from 40%–90% in literature (13, 14).

For those cases in which closed reduction has failed and in the case of failure of hip screening with late detected hip dislocation open reduction is often necessary (12).

The two most common open reduction approaches are the medially based approach or the anterior approach.

The use of the medial approach is limited for younger children because it limits the possibility to perform a concomitant pelvic osteotomy or capsulorrhaphy if necessary. Furthermore, the blood supply to the femoral head is more at risk and therefore the popularity of this approach has decreased (15).

Therefore, most authors prefer the modified Smith-Petersen anterolateral approach, that allows to perform a concomitant pelvic osteotomy and subsequent capsulorrhaphy (16).

Despite everything, there are still cases of severe developmental hip dislocation that fail treatment, even after open reduction. Re-dislocation after open reduction varies from 0% to 14% in literature (17, 18).

Orthopedic surgeons are unanimous that failure of open reduction is a serious complication and revision surgery appear to be technically demanding with high complication rates. Long-term consequences are usually serious (17).

Little attention has been given in literature to patients in whom open reduction of developmental hip dislocation has failed.

To our knowledge there are no guidelines when and how to perform revision surgery.

We therefore preformed a systematic review of the literature about current perspectives and timing when to perform surgical revision after failed open reduction of developmental hip dislocation in children.

## Methods

### Search strategy and selection criteria

Following the recommendations of the “Preferred Reporting Items for Systematic Reviews and Meta-Analyses” (PRISMA) statements we performed a comprehensive search of the PubMed bibliographic database and Google Scholar in order to select all studies published between 1980 and 2022.

Keywords and index terms (MeSH headings) used for the research in Pubmed and Google scholar were “Failed Open Reduction of Developmental Dysplasia of the Hip”; “Re-dislocation following open reduction for developmental dysplasia of the hip” and “Failed open reduction for congenital dislocation of the hip”.

Language was restricted to English, French and German. The “related articles function” was used to obtain any relevant reports. We manually reviewed the reference lists of identified studies for further inclusions. When duplicate studies were published with accumulating numbers of patients or increased duration of follow-up, only the one reporting the entire necessary outcomes was included. Eligibility was independently assessed by the authors. Extraction and data analysis were performed by each author in confirming the inclusion and exclusion criteria.

Studies were eligible for inclusion if they fulfilled the following criteria:

1) the study reported treatment of failed open reduction of developmental dislocation of the hip
2) age at surgery ranged between 1 months and 8 years

Studies reporting the treatment of developmental dislocation of the hip in children with any neuromuscular diseases (e.g., arthrogryposis multiplex congenital, cerebral palsy, spina bifida…) were excluded. Case reports, editorials, letters, and commentaries were excluded from the evidence review.

**Figure 1:**
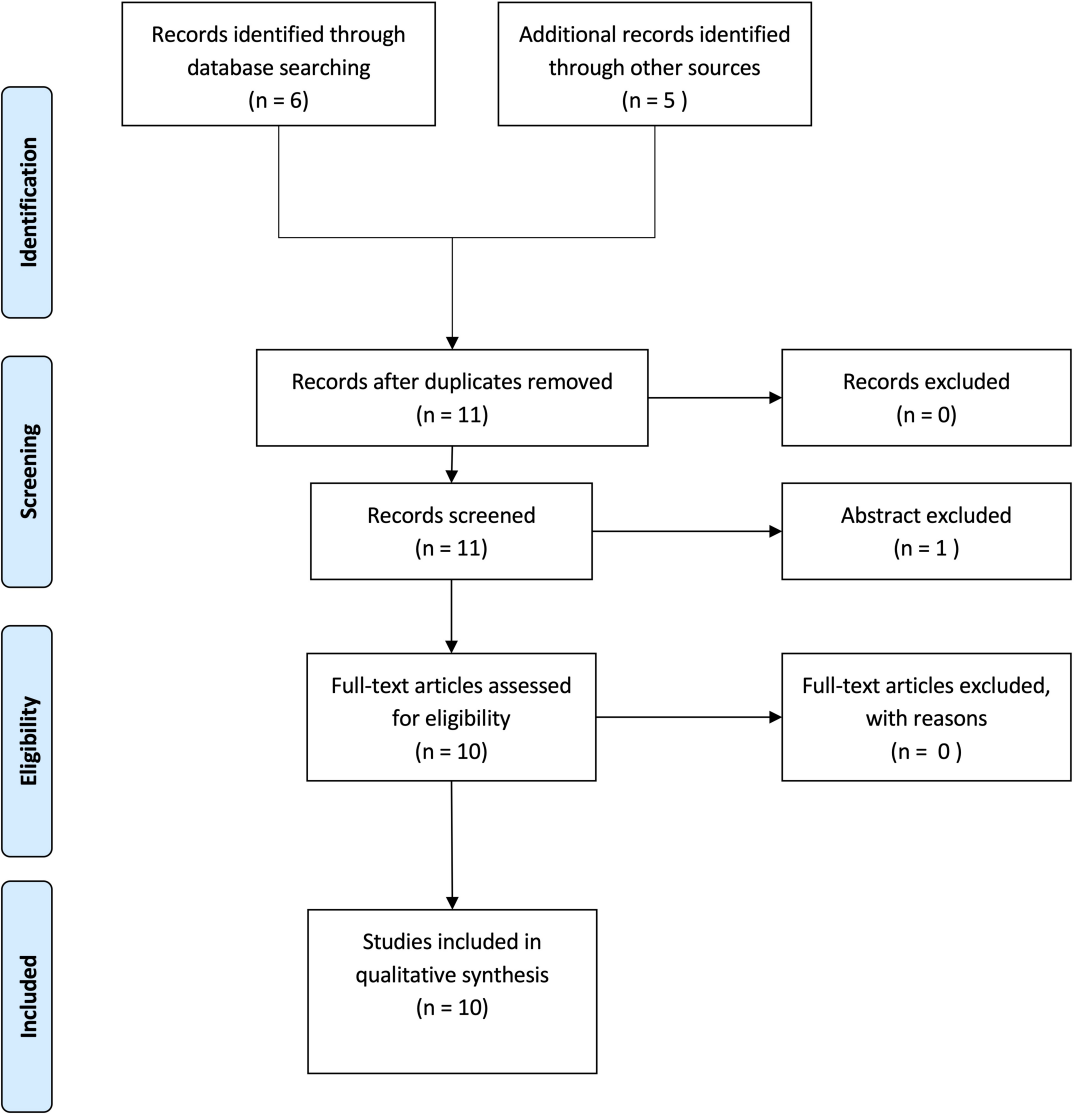
Flowchart diagram of the study selection process according to PRISMA.

The article selection process using a Preferred Reporting Items for Systematic Reviews and Meta-Analyses (PRISMA) flow diagram is presented in Figure 1, which included the studies reported further in Table 1.

**Table 1:** Literature overview and demographic data.

Author	Year	Number of hips	Number of patients	Timespan between OR and revision surgery (months)	Mean age at revision surgery (months)	Mean follow-up (year)	Approach
Bos et al. (19)	1984	15	14	13 (3–26)	36 (7–65)	10.6 (6–17)	Anterolateral (Smith-Peterson)
Herold et al. (20)	1983	35	27	n.r.	42 (24–42)	n.r.	Anterolateral (Smith-Peterson)
McCluske y (21)	1989	25	23	n.r	34 (11–45)	n.r.	Anterolateral (Smith-Peterson)
Kershaw et al. (22)	1993	33	32	n.r	43 (16–104)	6.4 (3–11)	Anterolateral (Smith-Peterson)
Hsieh et al. (23)	1998	34	32	8.9 (0–180)	69 (18–198)	3.9 (2–12.3)	Anterolateral (Smith-Peterson)
Chmielewski et al. (24)	2002	8	8	12.5 (3–27)	21.6 (12–34)	2.5 (0.5–4.4)	Anteromedial approach (Ludloff)
Kamath et al. (25)	2005	18	17	11 (0–45)	39 (24–72)	7.4 (2.5–16)	Anterolateral (Smith-Peterson)
Chidambaram et al. (26)	2006	14	14	25,5 (0–180)	69.4 (21–180)	1.7 (0.25–7)	Anterolateral (Smith-Peterson)
Abouelnas et al. (27)	2018	52	52	8.7 (6–14)	34.73 ± 12.69	4.2 (2–7.5)	Anterolateral (Smith-Peterson)
Elzohairy et al. (17)	2020	34	33	13 (6–30)	36 (24–54)	5.9 (5–7)	Anterolateral (Smith-Peterson)
Total/mean		268	252	13 (0–180)	35.5 (7–198)	4.8 (0.2–17)	90% Anterolateral approach

OR, open reduction; n.r, not reported.

Available demographic and treatments modalities were extracted from studies that met the inclusion criteria. Demographic data included number of patients, hips, age at revision surgery, timespan between open reduction, mean follow-up and revision surgery and the surgical techniques used for revision. Each study was then screened for clinical and radiological outcomes and complications as avascular necrosis of the femoral head, limb length discrepancy and redislocation rate.

Only case reports and small surgical series matched our inclusion criteria. Frequency and percentage were used for categorical data while mean and range were used for continuous data.

Owing to the high variation in study characteristics and heterogenicity of the series metanalysis of the data was not feasible.

## Results

### Literature search

Our search strategy retrieved a total of 10 articles, including 252 patients and 268 hips. Table 1 shows the retrieved articles, population characteristics and the surgical strategy.

Mean time span between failed open reduction and revision surgery was 13 months (min 0, max 180 months). Mean follow up was 4.8 years (2–16) (Table 1).

90% of the authors performed revision surgery through an anterolateral approach, combining pelvic and femoral osteotomies.

The most common reported causes for failure were inadequate soft tissue release, especially medial capsular release, and psoas tendon release.

Radiological results were reported by most authors according to the Severin classification.

Clinical outcome was either reported with the Ponseti classification or the McKay classification (28–30) (Table 2).

**Table 2:** Radiological and clinical outcomes according to the severin, ponseti and Mc Kay classifications (28–30).

Ponseti classification	
I	Asymptomatic
II	Slight hip pain after long walks
III	Limp, free motion and no pain
IV	Limp and limitation of motion, no pain
V	Limp and pain
VI	Limp, limitation of motion and pain
McKay classification	
Excellent	Stable painless hip, no limp, negative Trendelenburg sign, full range of movement
Good	Stable painless hip, slight limp, negative Trendelenburg sign, slight decrease in range of movement
Fair	Stable painless hip, limp, positive Trendelenburg sign, and limited range of movement, or a combination of these
Poor	Unstable or painful hip or both, positive Trendelenburg sign
Severin classification	
I	Normal
II	Moderate deformity of femoral head or neck or acetabulum
III	Dysplastic
IV	Subluxed
V	Articulating with secondary acetabulum
VI	Dislocated
VII	Arthritic

Out of a total of 268 hips, clinical outcome was reported in 200 hips. There were 59 hips with Ponseti grade I or an excellent McKay – score (29.5%), 92 hips with a Ponseti grade II, III or a good McKay score (46%), 38 hips with a Ponseti Grade IV and a fair McKay Score (19%) and finally only 11 hips with Ponseti V, VI and McKay score poor (5.5%).

Regarding the Severin classification, 225 hips were reported out of a total of 268 hips.

Grade I was present in 25 hips (11%), grade II in 113 hips (50%), grade III and IV in 68 hips (30%) and grade V, VI and VII in 19 hips (8.5%).

The complication rate for revision surgery after failed open reduction of congenital hip dislocation is high with avascular necrosis seen in 5% and 67% of cases and limb length discrepancy in 9%–47% of cases among study population. Redislocation rate after revision surgery varies from 6% to 13% (19, 21–23, 25, 26, 31).

Clinical and radiological results as well as reported complications are reported for each study in Table 3.

**Table 3:** Results after revision surgery of failed open reductions.

Author	Year	Number of hips	Number of patients	AVN	Complications	Radiological outcome (Severin)	Clinical outcome (Ponseti/Mc Kay)
Bos et al. (19)	1984	15	14	10 (67%)	Limb length discrepancy >2 cm (47%)	I (3)	I (6)
						II (6)	II (4)
						IV (4)	IV (4)
						V (2)	VI (1)
Herold et al. (20)	1983	35	27	n.r	n.r	n.r	n.r
McCluskey (21)	1989	25	23	11 (44%)	Limb length discrepancy >2 cm (30%)	I (3)	n.r
						II (9)	
						III (7)	
						IV (1)	
						VII (3)	
						n.r (2)	
Kershaw et al. (22)	1993	33	32	19 (58%)	Limb length discrepancy >2 cm (33%)	I (13)	I (16)
						II (9)	II (5)
						III (4)	III (6)
						IV (3)	IV (1)
						V (3)	V (3)
						VII (1)	VI (2)
Hsieh et al. (23)	1998	34	32	21 (62%)	Limb length discrepancy >2 cm (9%)	I (6)	Excellent (10)
						II (9)	Good (8)
						III (10)	Fair (11)
						IV (4)	Poor (3)
						VI (4)	
						VII (1)	
Chmielewski et al. (24)	2002	8	8	5 (63%)	n.r.	n.r	n.r
Kamath et al. (25)	2005	18	17	11 (61%)	Limb length discrepancy >2 cm (29%)	I (0)	I (4)
						II (12)	II (8)
						III (3)	III (1)
						IV (3)	IV (5)
Chidambaram et al. (26)	2006	14	14	7 (50%)	Limb length discrepancy >2 cm (36%)	I (0)	I (2)
						II (2)	II (1)
						III (10)	III (2)
						IV (1)	IV (8)
						VI (1)	VI (1)
Abouelnas et al. (27)	2018	52	52	14 (27%)	Limb length discrepancy >2 cm (36%)	I (0)	I (21)
						II (38)	II (22)
						III (11)	III (7)
						IV (3)	IV (2)
Elzohairy et al. (17)	2020	34	33	2 (5%)	Redislocation (2)	I (0)	Excellent (0)
					Sciatic nerve injury (1)	II (28)	Good (28)
						IV (4)	Fair (4)
						V (2)	Poor (2)
Total/mean	–	268	252	49%			

Complications, clinical and radiological results *(n.r. not reported, AVN: avascular necrosis).*

## Discussion

It is widely accepted, that the success of open reduction depends on the ability of the surgeon and in gentleness and accuracy with which the surgery is performed (16, 32). The most common causes of re-dislocation after open reduction are inadequate exposure and failure to release the obstructing soft tissues inside and around the hip, especially the psoas tendon, an insufficient capsular release, and a tight transverse ligament (17, 19, 23, 25, 27).

On the other hand, those structures, especially the psoas tendon, are close to the femoral neurovascular bundle, and therefore some surgeons may be too cautious when performing medial capsular release and psoas tendon release. Some authors reported that concomitant femoral or pelvic osteotomy can be a risk factor for subsequent posterior displacement of the femoral head (22).

Sankar et al. performed a matched cohort analysis about the risk factors for failure after open reduction for DDH. They conclude that bilateral involvement, greater pelvic width, and decreased abduction in the spica cast could be predictive of failure (33). Indeed, in some studies there was no evidence for failure in those patients presenting bilateral involvement, and it may be that the biological behavior of these hips is in some way different (25).

In several cases, dysplasia of the femoral head or an insufficiently corrected femoral version were thought to be the reason for the failure of the primary surgery (33).

Concentric reduction should be obtained at the second open reduction by addressing the causes of failure. This is best done by an anterolateral approach known as the Smith Peterson approach that was used in 90% of cases in our study (19, 21). It allows a good exposure of the adjacent soft-tissues and concomitant pelvic osteotomy and capsulorrhaphy when necessary (16). To obtain a satisfactory reduction and reduce pressure on the femoral head, femoral shortening is frequently due (21).

Acetabular insufficiency is often present, and most authors proceed to an acetabuloplasty at the same surgical time (19, 23, 25, 27).

Postoperatively the leg is hold in a pelvic spica cast for 6–12 weeks depending on subsequent pelvic or femoral osteotomies. After cast removal physiotherapy was introduced to regain range of motion (27). Another technique is the use of a Kirschner wire to stabilize the femoral head into the acetabulum after successful reduction. It can be performed with the common anterolateral approach. A reduced rate of re-dislocation was reported without an increase in avascular necrosis, triradiate cartilage growing arrest or septic arthritis (34, 35).

Timing when to perform revision surgery after the first open reduction is not clearly defined (19). Most authors agree, that after an initial failed open reduction, it is recommended to allow time for a regain of motion because hip stiffness is often present due to scarry tissues and cast (17, 21, 24, 25, 27).

Regarding clinical and radiological outcomes, some authors consider the age at definitive concentric reduction as the most predictive factor. Indeed, some authors showed a positive correlation between the age of the patient at revision surgery and clinical and radiological outcomes. Others correlated the outcome with the number of previous closed and open attempts of reduction (26, 27) and an age older than 4 was associated to a Severin score of 3 or worse (23).

Even if some authors could observe good functional results in hips with even type 4 femoral head necrosis, these functional results seem to deteriorate with the years, and it is evident that they will be worse after another follow-up period of 10 years (19).

We could observe a certain correlation between radiological and clinical outcome. But those data are to be interpreted with parsimony, as the studies present a great heterogenicity concerning follow-up and the use of different clinical classification systems as the Mckay score for some studies and the Ponseti score in others.

Another main factor of prognosis is avascular necrosis induced by previous treatment (32).

Indeed, the most common complication encountered in case of redislocation after failed open reduction of developmental hip dislocation is avascular necrosis of the femoral head with subsequent head subluxation (19, 21, 22). Literature reports between 37% and 68% of avascular necrosis of the femoral head after revision surgery of a failed open reduction (19, 21–23).

Whether AVN was due to revision surgery or the consequence of previous multiple attempts to reduce the hip is unclear. Most authors believe that AVN develops as a result of repeated surgeries leading to vascular damage of the femoral head and also due to the increased pressure after reduction (26).

Redisclocation after revision surgery varies from 6% to 13% (17, 19, 22, 23, 26). It is thought that a flattened and enlarged shape of the femoral head, a fairly consistent finding in patients that underwent revision surgery for failed open reduction as well as an increased anteversion, contributes to the re-dislocation risk (19). Therefore, surgeons should consider femoral derotation osteotomies as well as femoral shortening osteotomies in conjunction with repeated open reduction in order to minimize the risks of redisclocation and to ensure a concentric, stable and deep reduction of the femoral head into the acetabulum.

## Conclusion

Redislocation of developmental hip dislocation after an open reduction is a serious complication with poor long-term outcome mainly due to a high rate of avascular necrosis of the femoral head. Reasons for failure of the first open reduction are inadequate soft tissue release, especially an insufficient anteromedial capsular release. Prior to further open reduction, time should be allowed to regain some hip mobility before performing a secondary open reduction. It is mandatory to obtain a concentric, stable reduction at the second surgery combining soft tissue release, capsulorrhaphy, pelvic and femoral osteotomy if necessary. This is best done via an anterolateral approach.

Parents must be informed about the high risk of avascular necrosis and their consequences later in life.

The results of this systematic review must be interpreted with caution due to the small number of articles and the heterogeneity of the different studies included.
